# Validation of the Turkish Version of the Problem Areas in Diabetes Scale

**DOI:** 10.1155/2011/315068

**Published:** 2011-12-13

**Authors:** Elisabeth M. J. Huis In ‘T Veld, Ceylan Makine, Arie Nouwen, Çağatay Karşıdağ, Pinar Kadıoğlu, Kubilay Karşıdağ, François Pouwer

**Affiliations:** ^1^Department of Medical Psychology and Neuropsychology, Center of Research on Psychology in Somatic Diseases [CoRPS], Tilburg University, P.O. Box 90153, 5000 LE Tilburg, The Netherlands; ^2^School of Psychology, University of Birmingham, Birmingham B15 2TT, UK; ^3^Bakirkoy Mental and Neurological Disease Hospital, 34147 Bakırköy, Istanbul, Turkey; ^4^Cerrahpasa Medical Faculty, Istanbul University, 34098 Fatih, Istanbul, Turkey; ^5^Istanbul Medical Faculty, Istanbul University, 34452 Beyazit, Istanbul, Turkey

## Abstract

The Problem Areas in Diabetes (PAID) scale is a widely used self-report measure that can facilitate detection of diabetes-specific emotional distress in clinical practice. The aim of this study was to assess the factor structure and validity of the Turkish version of the PAID. A validation study was conducted among 154 patients with insulin-naïve type 2 diabetes. Participants completed the PAID, Centre for Epidemiological Studies Depression Scale (CES-D), Insulin Treatment Appraisal Scale (ITAS), and World Health Organization-Five Well-Being Index (WHO-5) questionnaires. Exploratory factor analyses yielded a 2-factor structure, identifying a 15-item “diabetes distress” factor and a 5-item “support-related issues” factor. The total PAID-score and the two dimensions were associated with higher levels of depression and poor emotional well-being. In the present study, the Turkish version of the PAID had satisfactory psychometric properties, however, the factorial structure was found to differ from factor solutions from other countries.

## 1. Introduction

Type 2 diabetes is an increasingly common, often burdensome illness. People living with type 2 diabetes not only have a chronic condition to cope with, but they are also faced with the presence and/or the prospect of serious complications [1]. The fact that living with diabetes often confronts patients with diabetes-specific stressors may explain the recent findings of Ali et al. [2], who found that depression is up to four times more likely in patients with type 2 diabetes compared to a general population. In depressed diabetes patients, increased levels of diabetes-specific emotional distress were found [3, 4]. Furthermore, diabetes-specific stressors were found to be associated with less adequate self-care and impaired glycemic control [5, 6]. However, the recognition rates of high levels of diabetes-specific emotional distress appeared to be low [7]. Therefore, it seems important that diabetes health care providers and diabetes researchers have access to instruments that can assess disease-specific emotional distress in a valid and reliable way. One widely used instrument is the Problem Areas in Diabetes (PAID) scale. The PAID is a 20-item self-report survey that assesses a range of emotional problems related to having type 1 or type 2 diabetes. Previous research has shown that the PAID is a clinically relevant and psychometrically sound instrument [8, 9]. Higher PAID scores were associated with lower levels of adherence to treatment and with higher levels of depression and HBA_1c_ [10, 11].

The PAID has been translated into several languages and has been validated in Hispanic [1], Dutch, American [8], Chinese [12], Brazilian [13], Swedish [14], Icelandic [15], African American [1, 16] samples. Recently, a 5-item version and a 1-item version of the PAID have been developed [17]. However, because the factor structure of the PAID seems to differ across cultures, further translation and cross-cultural validation of the PAID is needed. In 2002, the prevalence of diabetes in Turkey was estimated at 2.6 million. The aim of the present study is therefore to validate the Turkish version of the PAID and make this instrument available for use in Turkish diabetes research and clinical practices.

## 2. Participants and Methods

The study was conducted in two outpatient clinics in Istanbul, Turkey: the Istanbul Medical Faculty Hospital and the Cerrahpaşa Medical Faculty Hospital, as described by Makine et al. [18]. A total number of 220 patients with type 2 diabetes mellitus who were treated with diet and/or oral agents were invited to participate in the study. Exclusion criteria for this study were being illiterate or not being able to read due to vision problems. All participants gave written informed consent, and the Ethical Review Committee of the Istanbul University Medical Faculty and Cerrahpaşa Medical Faculty approved the study [18].

Table 1 shows the sample characteristics of the final 154 participants, separately for the two clinics. Mean age was 56 ± 10 years, mean duration of diabetes was 7 ± 6.5 years, and mean HbA_1c_ was 6.6 ± 1.0%.

### 2.1. Measures

Information was collected on age, sex, marital status, income, education, height, weight, time of diabetes onset, diabetes treatment, complications, and HbA_1c_ levels. Medical and psychological treatments and history of depression were collected using self-report. As a measure of concurrent validity of the PAID, patients were asked to rate their perception of the difficulty of regulating their diabetes (metabolic control) on a scale from 1 (not difficult) to 4 (very difficult). The Turkish versions of all measures were used. 

#### 2.1.1. Diabetes-Specific Emotional Distress (PAID)

The Problem Areas in Diabetes (PAID) scale is a self-report measure with 20 items and was translated by Makine et al. [18]. The items contain statements regarding four domains of quality of life with respect to diabetes (emotional distress, treatment issues, food-related problems, and lack of social support) and are rated on a scale from 0 (not a problem) to 4 (a serious problem). Scores on the PAID are summed and transformed to a 0–100 scale, with higher scores indicating greater emotional distress [8–11].

#### 2.1.2. Depression (CES-D)

The Centre for Epidemiological Studies Depression Scale (CES-D; [19, 20]) was used to assess the occurrence of depressive symptoms in the last week. The CES-D contains 20 items that are rated on a scale from 0 (rarely or never/less than one day) to 3 (most or all the time/5 to 7 days). CES-D scores are summed, with higher scores indicating more depressive symptoms. The internal consistency in this sample was 0.88.

#### 2.1.3. Insulin Treatment Appraisal Scale (ITAS)

The Insulin Treatment Appraisal Scale (ITAS; [18]) was used to assess attitudes towards insulin treatment [21]. The ITAS contains 20 items that are scored on a scale from 1 (strongly disagree) to 5 (strongly agree). The scores are summed, with a higher score indicating a more negative attitude towards insulin therapy. The internal consistency in this sample was 0.82.

#### 2.1.4. WHO-5

The World Health Organization-Five Well-Being Index (WHO-5; [22]) was used to assess general emotional well-being. The WHO-5 is a short 5-item measure, rated on a scale from 0 (not at all) to 5 (constantly) how often they have felt in a certain way in the last two weeks. The scores are summed and transformed to a 0–100 scale, with higher scores indicating better emotional well-being. The WHO-5 has an internal consistency of 0.87 in this sample.

### 2.2. Statistical Analysis

SPSS version 17 for Windows was used to carry out statistical analyses. Associations between categorical variables were analysed using nonparametric tests and continuous variables with *t*-tests. The Eigenvalue > 1 criterion and inspection of the scree plot [23] were used to determine the optimal number of factors. To assess the factor structure of the Turkish version of the PAID, exploratory factor analysis with oblimin rotation was used, as we assumed the factors to be correlated. Cronbach's *α* was calculated as a measure of internal consistency. To measure concurrent and convergent validity, Pearson product moment correlations, or *χ* ^2^ for dichotomous variables, were calculated. 

## 3. Results

### 3.1. Demographic Indices

Patients from the two clinics did not differ with regard to age, gender, diabetes duration, or treatment. However, the Cerrahpaşa University Hospital had a higher prevalence of depression (CES-D ≥ 16) than the Istanbul University Clinic (19% versus 32%, *ρ* < 0.05). Furthermore, depression appeared to be more common in women than in men with type 2 diabetes. Moreover, women had a significantly lower education level than men. 

Diabetes was treated with diet for 22.7% of the participants, 74,7% of the participants received oral medication and 2,6% of the participants did not receive any treatment yet. Mean value for the Turkish PAID was 27 ± 19. 

### 3.2. Factor Analysis

Using the Kaiser criterion (Eigenvalues > 1), a 1-, 2- or 3-factor solution could be calculated, but as a Dutch-US validation study found four dimensions, we also calculated the 4-factor solution (Table 2). The three- and four-factor solutions resulted in factors with low and double factor loadings. Interestingly, the two-factor solution resulted in a clear 15-item “diabetes distress” factor and a 5-item “support-related issues” factor. The eigenvalue of the diabetes distress factor was 10.4 (explaining 52% of the variance) and the eigenvalue of the support-related issues factor was 1.3 (explaining a further 6.7% of the variance before rotation). The item “feelings of deprivation regarding food” had the highest loading on the diabetes distress factor, and the item “feeling alone with your diabetes” loaded the highest on the support-related issues factor. The internal consistency of the PAID and the two factors were Cronbach's *α* = 0.85 for the support-related issues factor, Cronbach's *α* = 0.94 for the diabetes distress factor, and Cronbach's *α* = 0.95 for the total 20-item PAID.

### 3.3. Convergent Validity

Table 3 shows the correlations between total 20-item PAID, the two factors and other measures of interest. Also, correlations with the scores on the shorter versions of the PAID, the PAID-1, and PAID-5 are shown as suggested by McGuire et al. [17]. Higher scores on the total PAID and its subscales scores were significantly associated with higher levels of depression (CES-D) scores and a more negative appraisal of insulin therapy (ITAS scores), and also with higher ratings of having problems with the regulation of blood glucose levels. Negative associations were found with WHO-5 scores. Furthermore, older people had lower PAID scores. Additionally, the PAID scores appeared not to be associated with HbA_1c_ levels or the number of years since diagnosis. The results were comparable for the PAID-1 and PAID-5. 

Female participants had significantly higher total PAID scores than males (30 ± 19 versus 23 ± 19; *ρ* < 0.05), and women also scored slightly higher on both the diabetes distress factor (25 ± 15 versus 20 ± 15, *ρ* < 0.05) and support-related issues factor (5 ± 5 versus 3 ± 5, *ρ* < 0.05). Furthermore, a significant association with education was found: participants who attended primary school versus patients that attended college/high school reported higher levels of diabetes-specific emotional distress on the total PAID (35 ± 19 versus 22 ± 17, *ρ* < 0.05) and on the support-related issues factor (5 ± 5 versus 2 ± 3, *ρ* < 0.05). 

Moreover, patients who reported having complications (*n* = 57) scored significantly higher on the PAID total (31 ± 17 versus 24 ± 19, *P* < 0.05) and on the diabetes distress factor (27 ± 14 versus 20 ± 16, *ρ* < 0.05) than patients without complications. Also, patients with a history of depression treatment had significantly higher scores of diabetes distress (31 ± 14 versus 20 ± 15, *ρ* < 0.01), support-related issues (6 ± 6 versus 4 ± 4, *ρ* < 0.01), and total PAID scores (37 ± 18 versus 24 ± 17, *ρ* < 0.01).

## 4. Discussion

The aim of this study was to assess the psychometric properties of the Turkish version of the PAID. Exploratory factor analysis clearly yielded a solution with one general 20-item factor or two separate factors: “diabetes distress” and “support-related issues”. This is only partially consistent with findings from other studies that assessed the cross-cultural validity of the PAID. Factor analyses of the Swedish version of the PAID [14], consisting of 20 items, found a similar structure although with a three-factor solution: diabetes-related emotional problems, treatment-related problems, and also a support-related problems scale. Also, even though the factor solution in an Icelandic sample [15] resulted in two similarly named factors: “distress in relation to life with diabetes” and “distress in relation to management of diabetes”, they are not comparable to the factors found in this study.

In Dutch and US diabetes patients however, Snoek et al. [8] reported that both a one- and a four-factor solution (emotional, treatment, food, and lack of support problems) fitted well. Also, the Chinese version consisted of only one factor [12]. In short, even though the twenty item scales are comparable between countries, the subscales are not. Consequently, the comparability of study results from different countries is perhaps rather low in case subscales are being used. 

The Turkish version of the PAID and its two factors appeared to be internally consistent. As expected, the PAID and the two factors were strongly associated with generic measures of affect. Higher levels of diabetes distress were associated with a higher frequency of depressive symptoms occurrence, negative attitudes toward insulin treatment, difficulties with the regulation of the blood glucose, and lower levels of emotional well-being. Additionally, patients who reported having diabetes complications or a history of depression treatment scored significantly higher on the PAID, indicative of discriminative validity. Furthermore, PAID scores were unrelated to the number of years since diagnosis. Unexpectedly, and incongruent with other studies, the PAID was not related to HbA_1c_ levels [8, 10, 11, 14, 15]. However, the patients included in the sample were insulin naïve and well controlled with respect to HbA_1c_. The absence of poorly controlled patients in the present sample may thus explain why diabetes-specific emotional distress was not associated with glycemic control. A non significant association between PAID and HbA_1c_ was also found in a Brazilian sample (*n* = 146) [13] and two African American samples (*n* = 109 and *n* = 131) [1, 16]. These non significant associations may be explained by the small samples sizes of these studies. Additionally, an unexpected effect of education level on PAID scores was found, inconsistent with other studies [8–11] but in concurrence with some other studies [1, 13, 15]. The relationship between higher education and lower PAID scores may be explained by an increased understanding of HbA_1c_ knowledge, diabetes control, and diabetes care for patients with higher education [24]. 

Other studies have found a distress factor similar to the one in the current study. Interestingly, “worry about the future and the possibility of serious complications” was consistently found to be the emotional problem with the highest factor loading [8, 10, 11, 14, 16] whereas in the present study “feelings of deprivation regarding food” was the item with the highest loading. Furthermore, where a special “food-related problems” factor was found in the Dutch and US sample [8], in the current study, the items regarding food did not form a separate factor but instead were strongly integrated in the diabetes distress factor. These findings suggest cross-cultural differences in diabetes-related distress. Where in countries such as the Netherlands and the United States food issues are seen as related to diabetes but separate from social problems [8], it may be that in countries such as Turkey food is an integral and important factor of social life. A limitation of the study was that the sample was too small to allow confirmatory factor analysis, which could have provided with confirmatory evidence of the factor solution found in the current study. Furthermore, the test-retest reliability of the Turkish version was not assessed. 

In conclusion, the Turkish version of the PAID showed good internal consistency, discriminative, and convergent validity, but appeared to have a different factor structure than other versions. For international comparisons, use of the 20-item overall factor solution is recommended.

## Figures and Tables

**Table 1:** Demographic, clinical, and psychological characteristics of male and female participants with type 2 diabetes, who were treated in the outpatient clinic of the Istanbul University Hospital or the Cerrahpaşa University Hospital.

	Istanbul University Hospital		Cerrahpaşa University Hospital	
	Male	Female	Male	Female
*% (n)*	48% (47)	52% (51)	41% (23)	59% (33)
Age (years)	56 ± 10	55 ± 9	60 ± 13	56 ± 9
BMI	27 ± 3	30 ± 5	29 ± 4	31 ± 6
HbA_1c_ (mmol/mol)	6.8 ± 1.2 (51)	6.6 ± 0.9 (49)	6.3 ± 0.9 (45)	6.6 ± 0.9 (49)
Duration of diabetes (years)				
<1	9% (4/47)	2% (1/51)	26% (6/23)	15% (5/33)
1-2	15% (7/47)	16% (8/51)	9% (2/23)	15% (5/33)
3-4	15% (7/47)	20% (10/51)	13% (3/23)	15% (5/33)
5–10	38% (18/47)	41% (21/51)	35% (8/23)	30% (10/33)
>10	23% (11/47)	22% (11/51)	17% (4/23)	24% (8/33)
Level of education		*		*
Primary school	2% (1/47)	28% (14/51)	9% (2/23)	46% (15/33)
Middle school	15% (7/47)	6% (3/51)	13% (3/23)	24% (8/33)
College/High school	34% (16/47)	24% (12/51)	30% (7/23)	9% (3/33)
University	49% (23/47)	43% (22/51)	48% (11/23)	21% (7/33)
Diabetes complications				
No complication	60% (28/47)	63% (32/51)	70% (16/23)	64% (21/33)
Retinopathy	9% (4/47)	4% (2/51)	0% (0/23)	6% (2/33)
Cardiovascular	15% (7/47)	14% (7/51)	17% (4/23)	6% (2/33)
Nephropathy	2% (1/47)	6% (3/51)	0% (0/23)	6% (2/33)
Neuropathy	23% (11/47)	24% (12/51)	13% (3/23)	21% (7/33)
Foot problem	13% (6/47)	8% (4.51)	4% (1/23)	9% (3/33)
Mean CES-D score (depression)	9.7 ± 7.8	11.6 ± 8.9	7.3 ± 8.1	14.3 ± 13.0
High level of depression (CES-D ≥ 16)	13% (6/46)	24% (12/49)	22% (5/23)	40% (13/33)
Mean PAID total score (0–100)	28 ± 23	34 ± 22	30 ± 23	39 ± 24
Mean ITAS total score	52 ± 10	56 ± 9	54 ± 9	53 ± 13

*P ≤ 0.01, comparing men and women within the same hospital.

**Table 2:** 1-, 2-, 3-, and 4-factor solution after exploratory factor analysis of the 20 PAID items.

		Factor solution								
Shortened item content	1F	2F		3F			4F			
		F1	F2	F1	F2	F3	F1	F2	F3	F4
Feeling overwhelmed by your diabetes?	.87	.82		.73			.73			
Feeling discouraged with treatment plan?	.72	.56		.50			.34			.56
Feeling scared?	.82	.85		.74			.68			
Uncomfortable social situations?	.68	.86		.92			.89			
Feelings of deprivation regarding food?	.73	.89		.77			.82			
Feeling depressed?	.82	.88		.78			.75			
Feeling angry?	.85	.81		.74			.73			
Concerned about food and eating?	.81	.76		.70			.71			
Not “accepting” your diabetes?	.66	.50		.59			.44			.45
Mood related to your diabetes?	.80	.71		.41		.42	.40		.38	
Not having clear and concrete goals?	.5	.54				.67			.61	.51
Worrying about low blood sugar reactions?	.68	.60				.62			.59	
Coping with complications?	.70	.50				.51			.48	
Worrying about complications?	.70	.67				.85			.80	
Feelings of guilt or anxiety?	.68	.54				.78			.74	
Unsatisfied with diabetes physician?	.53		.69		.65			.65		
Diabetes is taking up too much energy?	.73		.74		.73			.75		
Feeling alone with your diabetes?	.73		.79		.81			.83		
Feeling that others are not supportive?	.54		.75		.72			.75		
Feeling “burned out”?	.76		.67		.66			.69		
Eigenvalue before rotation	10.4	10.4	1.3	10.4	1.3	1.2	10.4	1.3	1.2	0.9
% variance before rotation	52.1	52.1	6.7	52.1	6.7	6.2	52.1	6.7	6.2	4.5
Eigenvalue after rotation		9.9	7.0	8.9	6.6	6.8	8.6	6.9	5.9	2.0
Cronbach's *α*	.95	.94	.85	.94	.85	.86	.94	.85	.86	.67

**Table 3:** Correlations between total PAID, the two factors, the PAID-1 and PAID-5, and other measures of interest.

	CES-D	ITAS	WHO-5	Age	HbA_1c_	Diabetes Duration	Regulation Difficulty^†^
PAID total	.447**	.390**	−.472**	−.347**	.092	−.125	273.96^∗ ∗^
PAID factor 1 *Diabetes distress *	.412**	.367**	−.444**	−.348**	.083	−.126	258.17**
PAID factor 2 *Support-related issues *	.463**	.375**	−.454**	−.266**	.099	−.092	215.22**
PAID-1 * “Worrying about the future and the possibility of serious complications” *	.348**	.247**	−.309**	−.234**	.062	−.098	22.82*
PAID-5 *PAID items 3,6,12,16,19 *	.412**	.369**	−.414**	−.324**	.110	−.086	135.37**

*ρ ≤ 0.05

**ρ ≤ 0.01

^†^ *χ* ^2^ distribution.

## Abbreviations

PAID: Problem Areas In Diabetes survey
BMI: Body mass index
HbA_1c_: Glycated haemoglobin.
